# Real‐world evidence in health technology assessment of high‐risk medical devices: Fit for purpose?

**DOI:** 10.1002/hec.4575

**Published:** 2022-08-21

**Authors:** Philip Klein, Hedwig Blommestein, Maiwenn Al, Benedetta Pongiglione, Aleksandra Torbica, Saskia de Groot

**Affiliations:** ^1^ Institute for Medical Technology Assessment Erasmus University Rotterdam Rotterdam Netherlands; ^2^ Erasmus School of Health Policy & Management Erasmus University Rotterdam Rotterdam Netherlands; ^3^ Centre for Research on Health and Social Care Management (CERGAS) Bocconi University Milan Italy

**Keywords:** health technology assessment, high risk medical devices, observational studies of comparative effectiveness, real‐world‐evidence, reimbursement decision‐making

## Abstract

Health technology assessment (HTA) of medical devices (MDs) increasingly rely on real‐world evidence (RWE). The aim of this study was to evaluate the type and the quality of the evidence used to assess the (cost‐)effectiveness of high risk MDs (Class III) by HTA agencies in Europe (four European HTA agencies and EUnetHTA), with particular focus on RWE. Data were extracted from HTA reports on the type of evidence demonstrating (cost‐)effectiveness, and the quality of observational studies of comparative effectiveness using the Good Research for Comparative Effectiveness principles. 25 HTA reports were included that incorporated 28 observational studies of comparative effectiveness. Half of the studies (46%) took important confounding and/or effect modifying variables into account in the design and/or analyses. The most common way of including confounders and/or effect modifiers was through multivariable regression analysis. Other methods, such as propensity score matching, were rarely employed. Furthermore, meaningful analyses to test key assumptions were largely omitted. Resulting recommendations from HTA agencies on MDs is therefore (partially) based on evidence which is riddled with uncertainty. Considering the increasing importance of RWE it is important that the quality of observational studies of comparative effectiveness are systematically assessed when used in decision‐making.

## 1 INTRODUCTION

Health Technology Assessment (HTA) is used as a tool to support evidence‐based decision‐making and to ensure scarce resources are efficiently spent. Health technology assessment has been predominantly applied in pharmaceuticals. However, in the last decade, an increased interest has been noted for the application of HTA for other health technologies, such as medical devices (MDs) (Ciani, Federici, & Tarricone, 2016).

Traditionally, MDs need to adhere to less strict regulations to enter the market in the European Union compared with pharmaceuticals. They only need to meet the essential requirements for safety and performance meaning that the MD is safe and performs as intended (Council Directive 90/385/EEC; Council Directive 93/42/EEC; Directive 98/79/EC). Since 2007 the necessity has been recognized to enhance the clinical evaluation of implantable devices and class III (high risk) devices (Directive, 2007/47/EC). However, studies can be small clinical trials or non‐randomized clinical investigations (Tarricone, Torbica, & Drummond, 2017). It is unlikely that randomized controlled trials or other studies are setup after market approval, because the regulation of MDs already allows the use of MDs in clinical practice (Tarricone et al., 2016) and there are often no further requirements regarding the evidence of effectiveness. As a consequence evidence of effectiveness is often limited or not available at the start of an HTA (Enzing et al., 2021).

Another reason why evidence of effectiveness of high risk MDs from randomized controlled trials (RCTs) is not always available is that MDs are often subject to incremental innovations. Incremental innovations are defined as constant product modifications over the lifecycle of MDs. These modifications can impact the (cost‐)effectiveness of the MD (Ciani, Armeni, et al., 2016). As these modifications often entail small but continuous changes to the MD, Tarricone et al. argue that it is difficult to conduct RCTs at every single product development stage (Tarricone, Callea, et al., 2017). Real‐world evidence (RWE) collected along the lifecycle of a MD could help monitoring initial assumptions and recommendations made by continuously capturing incremental innovation over the lifecycle of the MD.

Real‐world evidence is used for different purposes in HTA, most importantly to estimate prevalence and/or incidence or to extrapolate short‐term effectiveness beyond trial duration, but also to evaluate safety, resource use, costs or quality of life (Makady et al., 2018). Additionally, RWE is used to evaluate comparative effectiveness of MDs to support HTA (Ciani et al., 2017; Makady et al., 2018). For example, in economic evaluations of implantable cardioverter defibrillators sources of treatment effects often consist of a synthesis of RCTs and observational studies (Tarricone, Callea, et al., 2017). Observational studies of comparative effectiveness are, however, prone to several types of bias (e.g., selection bias, information bias, recall bias, and detection bias (Blonde et al., 2018)). For example, when observational studies fail to take into account confounding and effect modifying variables in the design or analysis, this may result in biased estimates of the effectiveness and cost‐effectiveness of alternative health care interventions, which may lead to spending scarce resources sub‐optimally (Mitra & Indurkhya, 2005). Statistical methods to correct for confounding, such as regression, matching or instrumental variable (IV) estimation, provide unbiased estimates when the underlying assumptions are likely to be met (Kreif et al., 2013). However, very often these assumptions are not assessed, for example, studies fail to evaluate the “no unobserved confounding” assumption and do not consider the structural uncertainty from the choice of statistical approach (Kreif et al., 2013). Simultaneously, these statistical techniques are continuously being improved. For example, Genetic Matching extends propensity matching and may improve covariate balance (Sekhon & Grieve, 2012). Also methods to improve traditional IV methods are suggested (Basu et al., 2007). To what extent these methods are applied in practice to inform resource allocation, especially in the field of MDs is currently unknown.

The aim of this study was to evaluate the type of evidence of (cost‐)effectiveness used in HTA of high risk MDs in Europe and to assess the quality of RWE from observational studies of comparative effectiveness in particular. Although there have been indications that the quality of the evidence of (cost‐)effectiveness of MDs is lower compared to pharmaceuticals, (Fuchs et al., 2017; Olberg et al., 2017), this study extends the assessment of the quality of the evidence of effectiveness by using the Good Research for Comparative Effectiveness (GRACE) checklist. (Dreyer et al., 2016).

Recently, European Union (EU) regulation 2017/745 on MDs came into force that require MD manufacturers to obtain clinical evidence through an appropriate study design based on the latest scientific standards (Regulation (EU) 2017/745) In addition, clinical studies should be adequately powered to demonstrate the safety, performance and other aspects relating to benefit‐risk of devices. Our results provide insight in the quality of observational studies before the effects of the EU regulation 2017/745 come into play.

## 2 METHODS

Health technology assessment reports on high risk (class III) MDs from four European HTA agencies were selected: The National Institute for Health and Care Excellence (NICE; United Kingdom), the Health Information and Quality Authority (HIQA; Ireland), Zorginstituut Nederland (ZIN; The Netherlands), and the Agenzia Nazionale per i servizi sanitari Regionali (AGENAS; Italy). The aim was to include a selected group of HTA agencies which are known to conduct HTAs of MDs and that operate in different types of healthcare systems. As such, a broad geographical spread over the European region was not the (main) aim of the selection procedure nor did we aim to have a representative sample of all agencies in Europe. The selection resulted in a small but diverse group of European HTA agencies operating in different institutional contexts. The UK and Italy are, besides France and Germany, part of the four largest European jurisdictions with a large influence on European policies including policies concerning health (Makady et al., 2017). The Netherlands has, besides other countries, a pioneering role in European HTA projects (e.g., through the European network of HTA (EUnetHTA, n.d.). Language restrictions limited our selection to NICE, ZIN and AGENAS. NICE and ZIN are further known to accept real‐world data (RWD) regarding clinical effectiveness (Makady et al., 2017), while AGENAS has been particularly active in the evaluation of MDs. Ireland has been added because it is an example of one of the smaller EU member states with an HTA program covering MDs. Besides the reports from the HTA agencies of the countries discussed above, HTA reports on MDs published by EUnetHTA, a collaboration of HTA agencies across the EU, were selected.

The websites of the HTA agencies and EUnetHTA were searched for HTA reports on high risk MDs. The search strategy was informed by the *“Medical devices*: *Guidance document—Classification of medical devices”* published by the European Commission's Directorate General (DG) Health and Consumer (European Commission, DG Health and Consumer, 2010). Search terms were extracted from the descriptions of high risk MDs in this document. More details on the search strategy are provided in Appendix 1.

The following inclusion criteria were used to identify relevant HTA reports: (1) the assessment concerned high risk MDs, (2) assessments were considered only when they included some form of costs and outcomes evaluation, besides an assessment of clinical effectiveness, (3) in case of NICE, the medical technologies guidance (MTG) was selected, not NICE guidelines, diagnostics guidance, interventional procedures guidance and technology appraisal guidance. Also products from the NICE's advice programmes were not considered for inclusion (including the Medtech innovation briefings), as these do not have the status of formal NICE guidance (i.e., they contain no judgment on the value of the technology), and do not contain new NICE recommendations. We did not exclude any HTA reports based on publication date. The search was conducted in October‐November 2019.

Data were extracted from the HTA reports on the type of evidence demonstrating (cost‐)effectiveness. When RWE from observational studies of comparative effectiveness was presented in the HTA reports, the quality of this evidence was assessed. We followed the definitions of the Innovative Medicines Initiative GetReal project, that is, “RWE is the evidence derived from the analysis and/or synthesis of RWD”. Real‐world data is not collected in the context of an RCT, but instead from observations in clinical practice (prospectively and/or retrospectively) (Innovative Medicines Initiative, n.d.). To assess the quality of observational studies of comparative effectiveness, the individual studies were consulted (instead of using the information and assessment from the HTA report). The quality of the evidence was assessed using the GRACE checklist. This checklist is specifically designed to evaluate the quality of observational studies for decision‐making support. The GRACE checklist is an objective 11‐item checklist about data and methods covering the most important properties of high‐quality observational studies (Dreyer et al., 2016). When the aim of an observational study was to compare outcomes of two or more treatment alternatives, irrespective of whether this focused on the treatment effect, this study was included in our analysis.

## 3 RESULTS

In total, 25 HTA reports on high risk MDs were identified. Of these, 8 reports were published by NICE, 7 by HIQA, 2 by ZIN, 6 by AGENAS, and 2 by EUnetHTA. The MDs that were assessed varied from individual MDs (e.g., the Senza spinal cord stimulation system) to a class of MDs (e.g., robot‐assisted surgery and hip/knee prosthesis). Table 1 provides a summary of the key characteristics of the included HTA reports, and Table 2 provides an overview of the level of evidence presented in the HTA reports.

**TABLE 1:** Summary of key characteristics of included Health technology assessment (HTA) reports

	HTA reports (*n* = 25)
Publication date:	
<2010	5 (20%)
2010–2015	10 (40%)
2015–2019	10 (40%)
Country of publication:	
Ireland (HIQA)	7 (28%)
Italy (AGENAS)	6 (24%)
Netherlands (ZIN)	2 (8%)
United Kingdom (NICE)	8 (32%)
Other (EUnetHTA)	2 (8%)
Indication:	
Cardiovascular:	14 (56%)
Cerebrovascular	1 (4%)
Cardiac (CHD/AD)	9 (36%)
Peripheral arterial disease	4 (16%)
Orthopedic indication	6 (24%)
Other	6 (24%)

Abbreviations: AD, Arterial Disease; CHD, Chronic Heart Disease.

**TABLE 2:** Nature of evidence considered in Health technology assessment (HTA) reports and the policy recommendations

	HTA reports (*n* = 25)
Type of clinical studies:	
RCTs	9 (36%)
Real‐world data:	6 (24%)
Observational studies of comparative effectiveness	3/6 (50%)
Both RCTs and real‐world data:	10 (40%)
Observational studies of comparative effectiveness	9/10 (90%)
Type of economic evaluation:	
Cost minimization analysis	8 (32%)
Cost‐effectiveness analysis/Cost‐utility analysis	4 (16%)
(Systematic) review of cost‐effectiveness analyses	13 (52%)
Policy recommendation in HTA reports:	
Case supported by the evidence (NICE)	8 (32%)
Recommended	6 (24%)
Insufficient evidence/not recommended	5 (20%)
No advice regarding reimbursement or implementation^a^	6 (24%)

^a^ Purpose of EUnetHTA Core HTA reports is not to inform reimbursement decisions. These reports therefore do not include conclusions on whether the case of adopting the MD is supported.

### 3.1 Nature of evidence of effectiveness

In 19 (76%) reports evidence from RCTs was used. The evidence from RCTs differed in these reports, ranging from a single RCT to a meta‐analysis of numerous RCTs. The remaining 6 (24%) HTA reports were based on RWE only.

In total, 16 (64%) reports included some form of RWE. Real‐world evidence from observational studies of comparative effectiveness was presented in 12 (48%) reports including a total of 28 studies. These studies of comparative effectiveness differed in design, from cohort studies (non‐randomized) to observational studies derived from registries (i.e., registry‐based studies (Gliklich, Dreyer, & Leavy, 2014)) such as two studies included in the assessment of the transcatheter aortic valve implantation (TAVI) by AGENAS that used RWD from national registries to assess safety. Most of the observational studies of comparative effectiveness were part of the main body of evidence (20/28 [71%]) (Table 3). These studies were considered pivotal by the HTA agency in their assessment of the relevant treatment effect or other outcomes. For example, in the MTG on the ENDURALIFE powered CRT‐D devices, the external assessment center (EAC) assessed a selected number of publications submitted by the company, we considered those publications to be part of the main body of evidence. In contrast, the observational studies of comparative effectiveness within the reports by AGENAS on hip and knee prosthesis were considered as complementary evidence, because evidence from other sources was given more weight within these reports. Most studies were used to evaluate the relevant treatment effect of MDs (23/28 [82%]), but some were used for other purposes, for example, the evaluation of safety (Table 3).

**TABLE 3:** Relevance and purpose of observational studies of comparative effectiveness

	Main body of evidence	Complementary evidence
**Assessment of relevant treatment effect**	**NICE: ENDURALIFE powered CRT‐D devices** ‐ Alam et al., 2016(Alam et al., 2017) ‐ Ellis et al., 2016(Ellis et al., 2016) ‐ Landolina et al., 2015(Landolina et al., 2015) ‐ Von Gunten et al., 2015(von Gunten et al., 2016) **NICE: The MAGEC system** ‐ Akbarnia et al., 2013(Akbarnia et al., 2014) **HIQA: Robot‐assisted surgery (prostatectomy and hysterectomy)** ‐ Ficarra et al., 2009(Ficarra et al., 2009) ‐ Ball et al., 2006(Ball et al., 2006) ‐ Estape et al., 2009(Estape et al., 2009) ‐ Cardenas‐Goicoechea et al., 2010(Cardenas‐Goicoechea et al., 2010) ‐ Maggioni et al., 2009(Maggioni et al., 2009) ‐ Sarlos et al., 2010(Sarlos et al., 2010) **HIQA: Intermittent pneumatic compression** ‐ Chang et al., 2012(Chang et al., 2012) ‐ Kavros et al., 2008(Kavros et al., 2008) **ZIN: LVAD** ‐ Rogers et al., 2007(Rogers Joseph G. et al., 2007) **AGENAS: Implantable devices for the closure of patent foramen ovale** ‐ Thanopoulos et al., 2006(Thanopoulos, Dardas, Karanasios, & Mezilis, 2006) ‐ Windecker et al., 2004(Windecker Stephan et al., 2004) ‐ Schuchlenz et al., 2005(Schuchlenz et al., 2005) ‐ Harrer et al., 2006(Harrer et al., 2006) ‐ Casaubon et al., 2007(Casaubon et al., 2007)	**NICE: Senza spinal cord stimulation system** ‐ Tiede et al., 2013(Tiede et al., 2013) **AGENAS: Hip prosthesis** ‐ Sherfey et al., 2007(Sherfey & McCalden, 2006) **AGENAS: Knee prosthesis** ‐ Bozic et al., 2005(Bozic et al., 2005) ‐ Furnes et al., 2002(Furnes et al., 2002)
**Other use**	**EUnetHTA: MSCT coronary angiography** ‐ Schuijf et al., 2006(Schuijf Joanne D. et al., 2006)	**NICE: Senza spinal cord stimulation system** ‐ Van Buyten et al., 2017(Buyten et al., 2017) **NICE: The 3M Tegaderm CHG IV** ‐ Maryniak, 2009(Maryniak, 2009) **AGENAS: TAVI system** ‐ Bestehorn et al., 2015(Bestehorn et al., 2015) ‐ Brennan et al., 2017(Brennan J. Matthew et al., 2017)

*Note*: The bold text in this table represents first the HTA agency and second the name of the HTA dossier (typically the name of medical device).

### 3.2 Quality assessment of observational studies of comparative effectiveness

The results of the assessment of the quality of observational studies of comparative effectiveness used in the HTA reports using the GRACE‐checklist are presented in Table 4. In addition, Appendix 2 provides an overview of the titles and authors of each individual study and Appendix 3 provides the PICO‐TS of the studies (i.e., patient population, intervention, comparator, outcome, timing and setting).

**TABLE 4:** Quality assessment of observational studies of comparative effectiveness using the GRACE‐checklist

GRACE checklist items →	D1. Treatment (exposure) adequately recorded?	D2. Primary outcomes adequately recorded?	D3. Primary clinical outcome measured objectively?	D4. Primary outcomes validated, adjudicated, or otherwise known to be valid?	D5. Primary outcome measured in an equivalent manner between groups?	D6. Important covariates available and recorded?	M1. Population restricted to new initiators of treatment?	M2. Concurrent comparators?	M3. Confounding and effect modifying variables taken into account?	M4. Free of immortal time bias?	M5. Key assumptions tested?	Row sum +, *n* = 11 (%)	Part of the main body of evidence to assess the relevant treatment effect?
HTA reports & articles ↓													
NICE: Senza spinal cord stimulation system													
Tiede et al., 2013(Tiede et al., 2013)	**+**	**+**	‐	**+**	‐	‐	**+**	**+**	‐	**+**	‐	***55%***	***No***
Van Buyten et al., 2017(Buyten et al., 2017)	‐	‐	**+**	**+‐**	**+**	**+**	‐	**+**	**+**	**+**	‐	***55%***	***No***
NICE: ENDURALIFE powered CRT‐D devices													
Alam et al., 2017(Alam et al., 2017)	**+**	**+**	**+** ^a^	**+**	**+**	**+‐**	**+‐**	**+**	**+**	**+**	‐	***73%***	***Yes***
Ellis et al., 2016(Ellis et al., 2016)	**+**	**+**	**+** ^a^	**+**	**+**	**+**	‐	**+**	**+**	**+**	‐	***82%***	***Yes***
Landolina et al., 2015(Landolina et al., 2015)	**+**	**+**	**+** ^a^	**+**	**+**	**+**	‐	**+**	**+**	**+**	‐	***82%***	***Yes***
Von Gunten et al., 2016(von Gunten et al., 2016)	‐	**+**	**+** ^a^	**+**	**+**	‐	‐	**+**	‐	**+**	‐	***55%***	***Yes***
NICE: The 3M Tegaderm CHG IV													
*Maryniak*, *2009*(Maryniak, 2009)	**+**	**+**	**+** ^a^	‐	**+**	‐	‐	**+**	‐	**+**	‐	***55%***	***No***
NICE: The MAGEC system^c^													
*Akbarnia et al*., *2013*(Akbarnia et al., 2014)	‐	**+**	**+**	**+‐**	**+**	‐	**+**	**+**	**+**	**+** ^b^	‐	***64%***	***Yes***
HIQA: Robot‐assisted surgery* (prostatectomy and hysterectomy)													
Ficarra et al., 2009(Ficarra et al., 2009)	**+**	**+**	**+** ^a^	**+**	**+**	**+**	**+**	**+**	‐	**+**	‐	***82%***	***Yes***
Ball et al., 2006(Ball et al., 2006)	**+**	**+**	**+** ^a^	**+**	**+**	**+**	**+**	**+**	**+**	**+** ^b^	‐	***91%***	***Yes***
Estape et al., 2009(Estape et al., 2009)	**+**	**+**	**+**	**+‐**	**+**	**+‐**	**+**	**+**	‐	**+** ^b^	‐	***64%***	***Yes***
Cardenas‐Goicoechea et al., 2010(Cardenas‐Goicoechea et al., 2010)	**+**	‐	**+**	**+**	**+**	**+**	**+**	**+**	‐	**+** ^b^	‐	***73%***	***Yes***
Maggioni et al., 2009(Maggioni et al., 2009)	**+**	‐	**+**	**+**	**+**	**+**	**+**	‐	‐	**+** ^b^	‐	***64%***	***Yes***
Sarlos et al., 2010(Sarlos et al., 2010)	**+**	**+**	**+**	**+**	**+**	‐	**+**	‐	‐	**+** ^b^	‐	***64%***	***Yes***
HIQA: Intermittent pneumatic compression													
Chang et al., 2012(Chang et al., 2012)	‐	**+**	**+**	**+**	**+**	**+**	**+‐**	**+**	‐	**+** ^b^	‐	***64%***	***Yes***
Kavros et al., 2008(Kavros et al., 2008)	**+**	‐	**+**	**+**	**+**	**+**	**+‐**	**+**	‐	**+** ^b^	‐	***64%***	***Yes***
ZIN: LVAD													
*Rogers et al*., *2007*(Rogers Joseph G. et al., 2007)	**+**	**+**	**+**	**+**	**+**	**+**	**+**	**+**	‐	**+**	‐	***81%***	***Yes***
AGENAS: TAVI system													
Bestehorn et al., 2015(Bestehorn et al., 2015)	**+**	**+**	**+**	**+**	**+**	**+**	**+‐**	**+**	**+**	**+**	‐	***81%***	***No***
*Brennan et al*., *2017*(Brennan J. Matthew et al., 2017)	**+**	**+**	**+**	**+**	**+**	**+**	**+**	‐	**+**	**+**	**+**	***91%***	***No***
AGENAS: Implantable devices for the closure of patent foramen ovale													
Thanopoulos et al., 2006(Thanopoulos et al., 2006)	**+**	**+**	**+**	**+**	**+**	**+**	**+‐**	**+**	‐	**+**	‐	***73%***	***Yes***
*Windecker et al*., *2004*(Windecker Stephan et al., 2004)	**+**	**+**	**+**	**+**	**+**	**+**	**+‐**	**+**	‐	**+**	‐	***73%***	***Yes***
Schuchlenz et al., 2005(Schuchlenz et al., 2005)	**+**	**+**	**+**	**+**	**+**	**+**	**+‐**	**+**	**+**	**+**	‐	***81%***	***Yes***
Harrer et al., 2006(Harrer et al., 2006)	**+**	**+**	**+**	**+**	**+**	**+**	**+‐**	**+**	‐	**+**	‐	***73%***	***Yes***
Casaubon et al., 2007(Casaubon et al., 2007)	**+**	**+**	**+**	**+**	**+**	**+**	**+‐**	**+**	**+**	**+**	‐	***81%***	***Yes***
AGENAS: Hip prosthesis													
*Sherfey et al*., *2007*(Sherfey & McCalden, 2006)	**+**	**+**	**+**	**+**	**+**	**+**	**+**	**+**	‐	**+**	‐	***81%***	***No***
AGENAS: Knee prosthesis													
Bozic et al., 2005(Bozic et al., 2005)	**+**	**+**	**+**	**+**	**+**	**+**	**+**	**+**	**+**	**+**	‐	***91%***	***No***
Furnes et al., 2002(Furnes et al., 2002)	**+**	**+**	**+**	**+**	**+**	**+**	**+**	**+**	**+**	**+**	**+**	***100%***	***No***
EUnetHTA: MSCT coronary angiography													
*Schuijf et al*., *2006*(Schuijf Joanne D. et al., 2006)	**+**	**+**	**+**	**+**	**+‐**	**+**	**+**	**+**	**+**	**+** ^b^	‐	***81%***	***No***
**Column sum +, n = 28 (%)**	***86%***	***86%***	***96%***	***86%***	***93%***	***75%***	***50%***	***89%***	***46%***	***100%***	***7%***		

*Note*: (+) = Yes, (−) = No, (+‐) = Not enough information.

^a^ Not applicable as outcome is not clinical.

^b^ Not applicable.

^c^ The NICE guidance on the MAGEC system was withdrawn due to concerns about the safety of the device. Currently, it is advised to not use the MAGEC system until the safety of the device can be guaranteed.

Abbreviations: CHG IV, Chlorhexidine Gluconate Intravenous; CRT‐D, cardiac resynchronization therapy defibrillator; LVAD, left ventricular assist device; MAGEC, MAGnetic Expansion Control; MSCT, multi‐slice computed tomography; TAVI, Transcatheter Aortic Valve Implantation.

^*^ This report by HIQA on robot‐assisted surgery included over 80 observational studies. Due to this high number we assessed only those studies which were deemed of ‘high’ quality by HIQA’s quality appraisal.

#### 3.2.1 Items regarding data

In most of the observational studies of comparative effectiveness (24 out of 28, 86%), important information on the treatment was adequately recorded for the study purpose in the data sources. In the remaining four (14%) studies there was not enough information on the treatment, mainly because the data were not gathered in a study context but collected for medical purposes and documented in medical charts with insufficient details. For example, in the chart review by Van Buyten et al. (2017) data on a variety of spinal cord stimulation devices and technologies from various manufacturers were retrospectively extracted from medical charts from multiple implanting centers in three European countries (Buyten et al., 2017). Although this information on the treatment was not inherently reported for study purposes, the authors were able to divide the different devices and technologies into three distinctive groups. Explantation rates (i.e., any removal of an implantable pulse generator) associated to these devices and technologies, which is an important cost driver, were used to inform the cost model used by NICE to develop its guidance on the Senza spinal cord stimulation system.

In 24/28 (86%) observational studies of comparative effectiveness, the primary outcomes were adequately recorded for the study purpose. Similar to the recording of treatment, primary outcomes in the study of Van Buyten et al. (2017) were not deemed to be adequately recorded for the study purpose, that is, a limitation of this study was that the breakdown of explantation for reasons other than inadequate pain relief between device technologies were not fully reported (National Institute for Health and Care Excellence, 2019). Van Buyten et al. (2017) explain that the charts only provided information on general reasons for explant (Buyten et al., 2017). Also, 8% of implants were lost to follow‐up, for these it was unknown whether an explant had occurred elsewhere.

A total of 20 (71%) studies used a primary clinical outcome which was measured objectively. Of the remaining 8 (29%) studies, the primary outcomes were subject to the patient's opinion in one study. In this pilot study by Tiede et al. (2013), changes in pain intensity ratings were studied using a Visual Analog Scale, which was deemed a subjective outcome measure (Tiede et al., 2013). Although this study was considered by the EAC of NICE in its guidance on the Senza spinal cord stimulation system, this study was not considered to be relevant to the decision problem, possibly also because of its small sample size. Seven (25%) studies reported primary outcome(s) which were not of a clinical nature, thus this item of the GRACE checklist was not deemed applicable. The outcome in these studies was most often related to (incremental) improvements specific to MDs. For example, the entire comparative RWE base of NICE's report on the ENDURALIFE powered CRT‐D devices used battery life as the main primary outcome.

Most outcome measures were validated, adjudicated or otherwise deemed valid outcome measures. Only one study reported an outcome measure for which we had serious doubts about its validity. The evidence in the NICE report on the 3M Tegaderm CHG IV securement dressing included the nursing satisfactory assessment of Maryniak (2009) (Maryniak, 2009). The survey compared the intervention and comparator treatments using a 1–5 Likert scale. For example, nurses had to answer the question: “How was the overall performance of the CHG dressing (compared to a regular dressing)?” with 1 = much worse, to 5 = much better. As no additional information was provided about the validity of the employed methods of data collection in this study, we were not able to positively score this study. The study by Maryniak was not considered by the EAC as the study was not limited to critically ill patients and used non‐validated performance outcome measures.

In all but two (93%) studies the primary outcome was measured in an equivalent manner between (Maryniak, 2009) the intervention and comparator groups. In addition, the majority (*n* = 21; 75%) of studies reported known confounders and effect modifiers. However, the amount of detail provided varied greatly between studies from only a single sentence to several paragraphs dedicated to confounding. Five (18%) studies did not mention any known confounders, and for two (7%) studies some information was provided regarding confounders, however, it was not clear whether the most important known confounders were discussed mainly based on the fact that other studies reported more confounding variables in the same clinical setting.

#### 3.2.2 Items regarding methods

With regards to the employed methods, 14 of 28 (50%) observational studies of comparative effectiveness were conducted among a patient population that was restricted to new initiators of treatment. A further 9 of 28 (32%) studies did not substantiate on this topic, therefore we were unable to conclude whether patients were indeed restricted to new initiators or not. Furthermore, 25 of 28 (89%) studies comprised of concurrent comparator studies, meaning both the intervention and comparator group were evaluated within the same time frame. Only 3 of 28 (11%) studies used patient groups which were not concurrent, as was the case for the study by Maggioni et al. (2009) which was used as evidence of effectiveness in the report on robot‐assisted surgery by HIQA and compared prospectively collected data from 40 patients who underwent robotic radical hysterectomy to a historic cohort of 40 patients who received abdominal radical hysterectomy (Maggioni et al., 2009).

Even though most studies reported on known confounders and effect modifiers, only half of the studies (46%) took these confounding and/or effect modifiers into account in the design of the study or in the analyses. The most common way of including confounding and/or effect modifiers was through multivariable regression analysis. For example, in the study by Casaubon et al., 2007, which was considered by AGENAS in their report on implantable devices for the closure of patent foramen ovale, a multivariable Cox model was used to study the effect of treatment on recurrent stroke taking into account some important covariates (Casaubon et al., 2007). As acknowledged by the authors the number of patients per treatment group as well as the number of events was limited. As another example, Bestehorn et al., 2015 used matching to account for confounders and effect modifiers (Bestehorn et al., 2015). They exactly matched patients receiving either surgical aortic valve replacement (SAVR) or a transfemoral approach based on 10 variables describing the patients' risk and comorbidity profiles. In another study, Brennan et al., 2017 studied transcatheter‐versus SAVR using propensity score matching to account for confounders and effect modifiers (Brennan J. Matthew et al., 2017).

Overall, where relevant the study designs ensured that the classification of exposed and unexposed person‐time was free of immortal time bias.

Almost all of the studies (26 out of 28, 93%) did not conduct analyses to test key assumptions on which the study's results are reliant, such as the impact of varying exposure and/or outcome definitions (Dreyer et al., 2016). The only studies that did include meaningful additional analyses were Brennan et al. (2017) and Furnes et al. (2002), used in AGENAS reports on the TAVI system and Knee prosthesis respectively (Brennan J. Matthew et al., 2017; Furnes et al., 2002). The most elaborate of the two was Brennan et al. (2017) (Brennan J. Matthew et al., 2017). They conducted additional analyses to assess the influence of pre‐operative surgical risk on the outcomes of patients with aortic stenosis (Brennan J. Matthew et al., 2017). Although they did find a relationship between pre‐operative risk and clinical outcomes, the relative treatment effect (transcatheter aortic valve replacement vs. SAVR) was consistent over different levels of pre‐operative surgical risk.

Table 4 also shows the sum (in %) of positive scores on the GRACE checklist for each of the included observational study of comparative effectiveness. The quality of RWE varies greatly between studies and HTA reports. For example, the NICE HTA report on the Senza spinal cord stimulation system included two observational studies of comparative effectiveness which both yielded a score of 55% on the GRACE items, meaning that these studies are subject to multiple factors that reduce the quality of these studies. These studies were, however, not part of the main body of evidence to assess the relevant treatment effect. The HTA report on robot‐assisted surgery included a total of 82 observational studies, of which we assessed the six studies deemed of “high” quality by HIQA's own assessment of the evidence. The results of the GRACE assessment show that these studies were positively scored on 64%–91% of the items. It can be expected that the 76 observational studies deemed of lower quality by HIQA would score lower on the GRACE assessment as well. Although RWE was used as main body of evidence to determine the relevant treatment effect, the quality of this evidence seems to be hampered.

## 4 DISCUSSION

This paper sheds light on the quality of observational studies of comparative effectiveness that are used to inform HTA of MDs. We show that many of the HTA reports published by HTA agencies in Europe incorporated both evidence from RCTs and RWE from observational studies to establish the comparative effectiveness of MDs. A review of NICE appraisals between 2010 and 2016 of pharmaceuticals found that in 4% of the reports RWD were used to assess comparative effectiveness (Anderson et al., 2019). In our study on HTA in multiple countries we found that RWE was the only evidence in 24% of the reports. Our study confirms previous findings of differences in study designs of observational studies (Fuchs et al., 2017; Olberg et al., 2017), from cohort studies to registry‐based studies. A previous study found that the quality of the evidence of (cost‐)effectiveness of MDs is low given the study designs included in HTA reports (Olberg et al., 2017). This study extends the assessment of the quality of the evidence of effectiveness by using the GRACE checklist, and showed that evidence obtained from RWD often suffer from factors that reduce their quality and usefulness for decision‐making. Even though, we found that the clinical evidence is often not deemed of very high quality, it can still be deemed appropriate for decision‐making by HTA agencies in certain situations. In such situations one option would be to recommend the technology within the context of a coverage with evidence scheme.

The quality of the data and methods of the observational studies of comparative effectiveness that were included in the HTA reports deviated from good research practice and several studies had severe limitations. The areas of the GRACE checklist that most hampered the quality were related to the methodology employed in the studies. For example, important covariates, confounding, and effect modifying variables were very often not considered in the design or analysis. The studies which included confounding and/or effect modifiers did so mostly through multivariable regression analysis. Other methodologies were often not explored, such as propensity score matching or methodologies that adjust for unobserved confounders, such as IV methods. In addition, key assumptions were very rarely tested through additional analyses. As such, the impact of these assumptions, such as varying exposure and/or outcome definitions (Dreyer et al., 2016), on the results is often unknown.

Approaches to scoring the GRACE checklist do not yet exist. We believe not all criteria are relevant in every context, especially in those situations where the evidence was not used to assess the relevant treatment effect. However, some criteria are not met in situations where this is critical. For example, if the RWE concerned is the only evidence available or is part of the main body of evidence to determine effectiveness, adequately dealing with confounding and effect modifying variables is very important.

Due to the identified problems regarding the quality of observational studies of comparative effectiveness in HTA of MDs, it is questionable whether the effectiveness reported in these studies is in fact attributable to the MD itself, or whether other factors are driving differences in outcomes. That said, to our knowledge very few, if any, specific guidelines on the use of RWE in HTA of MDs have been published by European HTA agencies (Makady et al., 2017). Instead, there is a reliance on generic guidelines on the use of RWE in HTA (e.g., DSU NICE Technical Support Document 17 (Faria et al., 2015)), yet judging from the current study the evidence of effectiveness of MDs in HTA reports does not seem to adhere to these guidelines. It must be noted that the aim of the studies used as evidence of effectiveness in HTA was not always to inform decision‐making. For example, Akbarnia et al. (2014) concluded that their study emphasizes the need for longer follow‐up (Akbarnia et al., 2014). Nonetheless, this observational study by Akbarnia et al. was used as the main source of evidence of comparative effectiveness for the MAGnetic Expansion Control system. In more than one occasion, these types of studies were identified by HTA agencies and used as the main body of evidence to demonstrate (cost‐)effectiveness of MDs, which is concerning given the limitations of those studies. Ultimately, we would argue that the results of the current study show that a transparent quality assessment of the underlying evidence of policy recommendations is still broadly lacking.

This paper has some limitations; in general, there are limitations in what can be concluded from the analysis of HTA reports and the underlying evidence of effectiveness alone. Policy recommendations are often based on discussions that take place in the relevant HTA committees. These discussions may only partially based on the evidence of (cost‐)effectiveness as a wide range of other factors may often be considered. It is likely that many important features of these discussions are not adequately reflected in the published HTA reports. Therefore, the current study highlights only one particular, albeit important, part of the decision‐making process. These other factors are not taken into account in the current study, thus conclusions on to what extent the imperfections in the evidence of effectiveness affect recommendations and the role of these other factors have been missed. Further research should be conducted which includes these other factors which impact decision‐making, for example, by observing the relevant HTA committee meetings. Such research would be time‐intensive and was outside the scope of the current project. Despite the limitations noted above concerning the reality of making recommendations inevitably based on less than perfect evidence, we believe it is still important to document the imperfections in the evidence that decision‐makers have available when making recommendations regarding the reimbursement and use of MDs.

The study has a relatively small sample size as we were bound to include HTA agencies which publish their HTA reports in English, Italian or Dutch. As a consequence, we didn't include HTA reports from several bigger European HTA agencies such as Haute Autorité de santé (France) and Institut für Qualität und Wirtschaftlichkeit im Gesundheitswesen (Germany). In addition, we only included HTA agencies which publish their reports in the public domain. To widen the scope of our research to countries in Eastern Europe, we searched on the websites of AHTAPol (Poland), AAZ (Croatia), and JAZMP (Slovenia) but we were unsuccessful in finding any HTA reports in English. Last, we excluded all HTA reports on class I and II (a/b) MDs since it was expected that incorrect policy recommendations have the largest impact in class III devices.

In addition, we only considered assessments including some form of costs and outcomes evaluation besides an assessment of clinical effectiveness. The current study is part of an EU horizon 2020 project which focused on costs and outcomes of MDs, hence, we adhered to this focus in the current study. Although this criterion helped us to identify comprehensive assessments, it also led to us potentially missing some HTA reports for MDs which only included the assessment of the clinical effectiveness.

Because we selected HTA reports from NICE's medical technologies evaluation program (MTEP), we did not include the NICE TA on alternative prostheses for total hip replacement (TA304), while an assessment of arthroplasty of the hip by HIQA and an assessment of hip prosthesis by AGENAS were included in our analysis. From TA304 we know that confounding and effect modifying variables were considered in the comparison between hip resurfacing arthroplasty and total hip replacement (Faria et al., 2015). However, like AGENAS, these variables were not considered in the comparison between different types of total hip replacement. Only in sensitivity analyses, stratification by age and gender was applied to adjust for differences between patients receiving the different devices, but this analysis was limited to the number of potential confounders that were considered (Bell et al. 2016).

Although this analysis benefited from the inclusion of HTA reports from different HTA agencies, it was not possible to compare to what extent these agencies relied on RWE in HTAs of MDs. The agencies have different remits and tasks, and consequently the way they select technologies varies, for example, topics that are expected to have most benefit to patients and/or the health and social care system are selected for evaluation within the MTEP by NICE when they are likely to be cost saving or cost neutral. Most often NICE is notified by the manufacturer on technologies that could be relevant to evaluate. As a result, the technologies that were included in this analysis are very heterogeneous. Furthermore, the selection criteria for the MTEP, in particular the a priori expectations of the benefits of a technology, might explain why NICE recommended to adopt all 8 technologies that we included in our analysis.

Due to our lack of in‐depth technical and clinical knowledge regarding some of the MDs and related indications, we relied on the authors of the studies to mention that the outcome measures were in fact validated. In case there was still doubt, we reported that there was not enough information for us to make this judgment. Similarly, with regards to the question about whether confounders or effect modifiers were available and recorded, we relied on the authors to elaborate on known confounders and effect modifiers. In case there were doubts about whether all confounders and effect modifiers were discussed, we reported that there was not enough information to make this judgment. This emphasizes the need for study reporting to be improved, consistent with the GRACE checklist.

## 5 CONCLUSION

In the past decade, we have witnessed greater use of RWE with the explicit goal of strengthening the evidence base of MDs for both the approval and reimbursement processes. Health technology assessment agencies have opened their doors to RWE, to different extents, but there are still important limitations in the quality of the RWE used, notwithstanding methodological advancements in the field. It is of utmost importance for the HTA agencies to review available methodologies and critically assess the evidence base with the explicit aim to strengthen the quality of RWE underpinning their recommendations. We do not necessarily argue for a more general adoption of the GRACE checklist, but we do believe that it is important that the quality of observational studies of comparative effectiveness are systematically assessed when used in decision‐making and that there is particular room for improvement regarding accounting for important confounding and effect modifying variables and in testing key assumptions.

## CONFLICT OF INTEREST

The authors report grants from European Union's Horizon 2020 research and innovation program under grant agreement #779306, during the conduct of the study.

## Supporting information

Supporting Information S1

Supporting Information S2

Supporting Information S3

## Data Availability

The data that support the findings of this study are openly available at the websites of the HTA agencies.
