# Supplementary material for: Real‐world evidence in health technology assessment of high‐risk medical devices: Fit for purpose?
Source: Health Econ. 2022 Aug 21;31(Suppl 1):10–24. doi: 10.1002/hec.4575 (PMC9541731; doi:10.1002/hec.4575)
Supplement: Supplementary file 1 — Supporting Information S1 [file HEC-31-10-s003.docx]

# Appendix 1

Typically, HTA agencies use separate recognisable coding for HTA reports on MDs as compared to pharmaceuticals (e.g. NICE uses the medical technologies guidance [MTG] code). If this was the case, we used this code to identify relevant reports. In case such a code was not used for MD reports, we created a search strategy to guide our search. This search strategy was informed by the *‘MEDICAL DEVICES: Guidance document – Classification of medical devices’* document published by the European Commission’s DG Health and Consumer.

Search terms were extracted from the tables on class III devices (e.g. the table on p. 35 and p. 37), these search terms* included:

**Table X.X. Search terms class III MDs**

| *Medical Technology* | *Arthroplasty* | *Shunt* |
| --- | --- | --- |
| *Technology* | *Device* | *Probe* |
| *Mechanical* | *Defibrillation* | *Drain* |
| *Catheter* | *Implant* | *Electrode* |
| *Pacemaker* | *Dialysis* | *Sutures* |
| *Invasive* | *(Neuro) endoscope* | *biological adhesive* |
| *Robot* | *(Brain) spatula* | *prosthetic/ prosthesis* |
| *Stent* | *Stimulation* | *aneurysm clip* |
| *Knee* | *Retractor* | *Pump* |
| *hip* | *spinal needle* | *joint replacement* |

*terms were translated for the search in the Dutch database from ZIN as well as in our search for reports by the Italian HTA agency AGENAS

AGENAS does not have a separate search function on their website to find HTA reports in a systematic way. Hence, we conducted a search for the Italian HTA agency on Google using Italian translations of the terms presented in the previous table.
