# Supplementary material for: Real‐world evidence in health technology assessment of high‐risk medical devices: Fit for purpose?
Source: Health Econ. 2022 Aug 21;31(Suppl 1):10–24. doi: 10.1002/hec.4575 (PMC9541731; doi:10.1002/hec.4575)
Supplement: Supplementary file 2 — Supporting Information S2 [file HEC-31-10-s001.docx]

Appendix 2

| **HTA agency** | **HTA report** | **Observational studies of comparative effectiveness** |
| --- | --- | --- |
| **NICE** | Senza spinal cord stimulation (SCS) system (Nevro) | Tiede, J., Brown, L., Gekht, G., Vallejo, R., Yearwood, T., & Morgan, D. (2013). Novel spinal cord stimulation parameters in patients with predominant back pain. Neuromodulation: Technology at the Neural Interface, 16(4), 370-375. |
|  |  | Van Buyten, J. P., Wille, F., Smet, I., Wensing, C., Breel, J., Karst, E., ... & Vesper, J. (2017). Therapy‐related explants after spinal cord stimulation: results of an international retrospective chart review study. Neuromodulation: Technology at the Neural Interface, 20(7), 642-649. |
|  | ENDURALIFE powered CRT-D devices | Alam, M. B., Munir, M. B., Rattan, R., Adelstein, E., Jain, S., & Saba, S. (2017). Battery longevity from cardiac resynchronization therapy defibrillators: differences between manufacturers and discrepancies with published product performance reports. Ep Europace, 19(3), 421-424. |
|  |  | Ellis, C. R., Dickerman, D. I., Orton, J. M., Hassan, S., Good, E. D., Okabe, T., ... & Greenspon, A. J. (2016). Ampere hour as a predictor of cardiac resynchronization defibrillator pulse generator battery longevity: a multicenter study. Pacing and Clinical Electrophysiology, 39(7), 658-668. |
|  |  | Landolina, M., Curnis, A., Morani, G., Vado, A., Ammendola, E., D'onofrio, A., ... & Gasparini, M. (2015). Longevity of implantable cardioverter-defibrillators for cardiac resynchronization therapy in current clinical practice: an analysis according to influencing factors, device generation, and manufacturer. Ep Europace, 17(8), 1251-1258. |
|  |  | Von Gunten, S., Schaer, B. A., Yap, S. C., Szili-Torok, T., Kühne, M., Sticherling, C., ... & Theuns, D. A. (2016). Longevity of implantable cardioverter defibrillators: a comparison among manufacturers and over time. Ep Europace, 18(5), 710-717. |
|  | The 3M Tegaderm CHG IV securement dressing | Maryniak, K. (2009). Clinical performance and nursing satisfaction of a transparent chlorhexidine gluconate IV securement dressing with peripherally inserted central catheters. Journal of the Association for Vascular Access, 14(4), 200-203. |
|  | MAGEC system | Akbarnia, B. A., Pawelek, J. B., Cheung, K. M., Demirkiran, G., Elsebaie, H., Emans, J. B., ... & Growing Spine Study Group. (2014). Traditional growing rods versus magnetically controlled growing rods for the surgical treatment of early-onset scoliosis: a case-matched 2-year study. Spine Deformity, 2(6), 493-497. |
| **HIQA** | Robot-assisted surgery | Ficarra, V., Novara, G., Fracalanza, S., D’Elia, C., Secco, S., Iafrate, M., ... & Artibani, W. (2009). A prospective, non‐randomized trial comparing robot‐assisted laparoscopic and retropubic radical prostatectomy in one European institution. BJU international, 104(4), 534-539. |
|  |  | Ball, A. J., Gambill, B., Fabrizio, M. D., Davis, J. W., Given, R. W., Lynch, D. F., ... & Schellhammer, P. F. (2006). Fourth Prize: Prospective Longitudinal Comparative Study of Early Health-Related Quality-of-Life Outcomes in Patients Undergoing Surgical Treatment for Localized Prostate Cancer: A Short-Term Evaluation of Five Approaches from a Single Institution. Journal of endourology, 20(10), 723-731. |
|  |  | Estape, R., Lambrou, N., Diaz, R., Estape, E., Dunkin, N., & Rivera, A. (2009). A case matched analysis of robotic radical hysterectomy with lymphadenectomy compared with laparoscopy and laparotomy. Gynecologic oncology, 113(3), 357-361. |
|  |  | Cardenas-Goicoechea, J., Adams, S., Bhat, S. B., & Randall, T. C. (2010). Surgical outcomes of robotic-assisted surgical staging for endometrial cancer are equivalent to traditional laparoscopic staging at a minimally invasive surgical center. Gynecologic oncology, 117(2), 224-228. |
|  |  | Maggioni, A., Minig, L., Zanagnolo, V., Peiretti, M., Sanguineti, F., Bocciolone, L., ... & Vélez, J. I. (2009). Robotic approach for cervical cancer: comparison with laparotomy: a case control study. Gynecologic oncology, 115(1), 60-64. |
|  |  | Sarlos, D., Kots, L., Stevanovic, N., & Schaer, G. (2010). Robotic hysterectomy versus conventional laparoscopic hysterectomy: outcome and cost analyses of a matched case–control study. European Journal of Obstetrics & Gynecology and Reproductive Biology, 150(1), 92-96. |
|  | Intermittent pneumatic compression | Chang, S. T., Hsu, J. T., Chu, C. M., Pan, K. L., Jang, S. J., Lin, P. C., ... & Huang, K. C. (2012). Using intermittent pneumatic compression therapy to improve quality of life for symptomatic patients with infrapopliteal diffuse peripheral obstructive disease. Circulation Journal, 1201311575-1201311575. |
|  |  | Kavros, S. J., Delis, K. T., Turner, N. S., Voll, A. E., Liedl, D. A., Gloviczki, P., & Rooke, T. W. (2008). Improving limb salvage in critical ischemia with intermittent pneumatic compression: a controlled study with 18-month follow-up. Journal of vascular surgery, 47(3), 543-549. |
| **ZIN** | Left Ventricular Assist Device (LVAD) | Rogers, J. G., Butler, J., Lansman, S. L., Gass, A., Portner, P. M., Pasque, M. K., ... & INTrEPID Investigators. (2007). Chronic mechanical circulatory support for inotrope-dependent heart failure patients who are not transplant candidates: results of the INTrEPID Trial. Journal of the American College of Cardiology, 50(8), 741-747. |
| **AGENAS** | Edwards SAPIEN 3 and Medtronic Evolut R | Bestehorn, K., Bestehorn, M., & Fleck, E. (2015). Influence of different approaches of aortic valve replacement on the incidence of post-operative delirium in intermediate risk patients–a matched pair analysis. Current medical research and opinion, 31(12), 2157-2163. |
|  |  | Brennan, J. M., Thomas, L., Cohen, D. J., Shahian, D., Wang, A., Mack, M. J., ... & Peterson, E. D. (2017). Transcatheter versus surgical aortic valve replacement: propensity-matched comparison. Journal of the American College of Cardiology, 70(4), 439-450. |
|  | Implantable devices for PFO closure | Thanopoulos, B. V. D., Dardas, P. D., Karanasios, E., & Mezilis, N. (2006). Transcatheter closure versus medical therapy of patent foramen ovale and cryptogenic stroke. Catheterization and cardiovascular interventions, 68(5), 741-746. |
|  |  | Windecker, S., Wahl, A., Nedeltchev, K., Arnold, M., Schwerzmann, M., Seiler, C., ... & Meier, B. (2004). Comparison of medical treatment with percutaneous closure of patent foramen ovale in patients with cryptogenic stroke. Journal of the American College of Cardiology, 44(4), 750-758. |
|  |  | Schuchlenz, H. W., Weihs, W., Berghold, A., Lechner, A., & Schmidt, R. (2005). Secondary prevention after cryptogenic cerebrovascular events in patients with patent foramen ovale. International journal of cardiology, 101(1), 77-82. |
|  |  | Harrer, J. U., Wessels, T., Franke, A., Lucas, S., Berlit, P., & Klötzsch, C. (2006). Stroke recurrence and its prevention in patients with patent foramen ovale. Canadian journal of neurological sciences, 33(1), 39-47. |
|  |  | Casaubon, L., McLaughlin, P., Webb, G., Yeo, E., Merker, D., & Jaigobin, C. (2007). Recurrent stroke/TIA in cryptogenic stroke patients with patent foramen ovale. Canadian journal of neurological sciences, 34(1), 74-80. |
|  | Hip prosthesis | Sherfey, J. J., & McCalden, R. W. (2006). Mid-term results of Exeter vs Endurance cemented stems. The Journal of arthroplasty, 21(8), 1118-1123. |
|  | Knee prosthesis | Bozic, K. J., Kinder, J., Menegini, M., Zurakowski, D., Rosenberg, A. G., & Galante, J. O. (2005). Implant survivorship and complication rates after total knee arthroplasty with a third-generation cemented system: 5 to 8 years followup. Clinical Orthopaedics and Related Research (1976-2007), 430, 117-124. |
|  |  | Furnes, O., Espehaug, B., Lie, S. A., Vollset, S. E., Engesæter, L. B., & Havelin, L. I. (2002). Early failures among 7,174 primary total knee replacements: a follow-up study from the Norwegian Arthroplasty Register 1994-2000. Acta Orthopaedica Scandinavica, 73(2), 117-129. |
| **EUnetHTA** | MSCT coronary angiography | Schuijf, J. D., Wijns, W., Jukema, J. W., Atsma, D. E., de Roos, A., Lamb, H. J., ... & Bax, J. J. (2006). Relationship between noninvasive coronary angiography with multi-slice computed tomography and myocardial perfusion imaging. Journal of the American College of Cardiology, 48(12), 2508-2514. |
