# Supplementary material for: Real‐world evidence in health technology assessment of high‐risk medical devices: Fit for purpose?
Source: Health Econ. 2022 Aug 21;31(Suppl 1):10–24. doi: 10.1002/hec.4575 (PMC9541731; doi:10.1002/hec.4575)
Supplement: Supplementary file 3 — Supporting Information S3 [file HEC-31-10-s002.docx]

# Appendix 3

|  | **P** | **I** | **C** | **O** | **T** | **S** |
| --- | --- | --- | --- | --- | --- | --- |
| Tiede et al. 2013 | Patients with chronic axial back pain eligible for spinal cord stimulation trial | Portable, external trial stimulator (ETS) similar to other externalized devices worn outside of the body during the temporary trial phase. The waveform was biphasic and charge-balanced. All patients received 30 msec stimulation pulses delivered at a rate of 10,000 Hz. Stimulation current ranged from 0.5 to 5.0 mA. | a typical SCS “Trial Phase” with a commercially available ETS to determine if the patient’s response was appropriate to continue on to a permanent, implantable SCS device. | Pain intensity ratings, subjective descriptions, and patients’ preference. | During trial (external stimulator) usual care 4-7 days, intervention 4 days | Hospital |
| Van Buyten et al. 2017 | Patients with chronic pain requiring dorsal column stimulation | High-frequency rechargeable SCS | Conventianal SCS, rechargable and non-rechargeable | Explant (for inadequate pain relief) | Median follow-up was 2.24 year | Hospital (2NL, 1 Belgium, 1 German) |
| Alam et al. 2016 | Patients with heart failure requiring CRT defibrilator | All CRT-D, 3 brands | see I | time from device implantation to battery depletion | Mean follow-up 3.4 year | Hospital seeting - scanned device printouts |
| Ellis et al. 2016 | Patients with a CRT-D device | CRT-D's with 1.0 Ah, 1.4 Ah, and 2.0 Ah battery capacity | see I | CRT-D survival (from implant time to time replacement, heart transplant, device infection, patient death or end-of-study) | Average follow-up 4 years | Hospital setting |
| Landolina et al. 2015 | Patients with a CRT-D device | CRT-D's (5 company's, 18 different devices/batteries) | See I | rate of replacement for battery depletion (service life from implantation to replacement, censored at death, last visit, removals for other causes) | Median follow-up was 43 months/3.6 years | Hospital setting |
| Von Gunten et al. 2015 | Patients with ICD (single chamber, dual chamber, CRT-D) | All ICDs (7 manufacturers, VVI, DDD and CRT-D) | See I | time from implant to replacement for battery depletion (service life from implantation to replacement, censored at death, last visit, removals for other causes) | Median follow-up was 53 months/4.4 years | Hospital setting |
| Maryniak et al. 2009 | Patients with Piripherally inservted central venous catheters | 3M Tegaderm CHG IV Securement Dressing | IV3000 (Smith & Nephew) | Nurses' satisfaction with overall performance of CHG dressing | Each dressing can stay on for a maximum of 7 days, and then needs to be changed. Hospital (in and out patient) |  |
| Akbarnia et al. 2013 | Progressive early-onset scoliosis (EOS) patients who met the following inclusion criteria: aged 10 years or younger at the time of index MCGR surgery, major curve 30° or greater, radiographic thoracic height (T1eT12) less than 22 cm, no previous spine surgery, and minimum 2-year postoperative follow-up. Indications for surgical intervention included severity of curve magnitude, rate of curve progression, and preservation of pulmonary function and who had complete data available for analysis. | A magnetically controlled growing rod (MCGR) was developed to reduce the number of surgical procedures and lessen the burden of repeated surgeries for EOS patients. | Traditional growing rod (TGR) surgery requires periodic surgical lengthenings of the construct, typically every 6 months, to maintain curve correction and allow continued spinal growth. | Spinal height (T1-S1) was measured on posteroanterior radiographs from the midpoint of the cephalad end plate of T1 to the midpoint of the cephalad end plate of S1. The vertical distance between the midpoint of the end plates was measured as T1-S1.  Thoracic height (T1-T12) was measured identically; however, the caudal reference point was the midpoint of the caudal end plate of T12. To eliminate the effect of index surgery on the increase in spinal height as well as the differences in length of follow-up between patients, the researchers calculated annual T1-S1 growth, defined as the change in spinal height (in millimeters) during the lengthening period (after index surgery to latest follow-up) divided by the duration of time in years.  Annual T1-T12 growth was calculated using the same formula to assess changes in thoracic height during the lengthening period. A single observer independently reviewed radiographs to verify the accuracy of measurements. | The following time points were analyzed: before surgery to immediately after surgery, immediately after surgery to latest follow-up (lengthening period), and before surgery to latest follow-up. Mean postoperative follow-up was 2.5 years (range, 1.9-3.0 years) for MCGR patients and 4.1 years (range, 1.8-6.9 years) for TGR patients. | Hospitals: Five international institutions. |
| Ficarra et al. 2009 | Patients undergoing prostatectomy for clinically localized prostate cancer | Robot-assisted laparoscopic prostatectomy (RALP) | Retropubic radical prostatectomy (RRP) | clinical and pathological variables: age, body mass index (BMI), Eastern Cooperative Oncology Group (ECOG) performance status, Charlson score, preoperative total PSA level, Gleason score at biopsy, clinical stage (cT, TNM 2002), Gleason score at RP, pathological stage (pT) and positive surgical margin (PSM) rate. perioperative variables: surgical time for RP, blood loss, perioperative transfusion rate, complications (during surgery, and early, within the first 30 days after surgery), status of the vesicourethral anastomosis at cystography, time to catheter removal and hospital stay. functional outcomes: urinary continence at the time of catheter removal, urinary continence at the 12-month follow-up, time to recovery of urinary continence, presence of bladder neck stenosis; erectile function at the 12-month follow-up, time to recovery of erectile function. | Clinical and pathological variables were collected prospectively.  Questionnaires before surgery and at 12 months follow-up | Hospital: Enrolment from February 2006-April 2007 at Urology Clinic, University of Padua Italy |
| Ball et al. 2006 | Patients with newly diagnosed clinically localized prostate cancer candidate for surgical intervention. | Laparoscopic radical prostatectomy (LRP), da Vinci robotic prostatectomy (dVP), brachytherapy (Pd) and prostate cryoablation (Pcryo) | Open radical prostatectomy (ORP) | UCLA PCI (disease-specific HRQoL) and AUA SI (obstructive and irritative symptoms) | Patients were asked to complete a pre-treatment survey and at 1, 3, 6, 9, 12, 18, 24, and 36 months to track longitudinal changes | Hospital: Patients seen from January 2000 - April 2005 at the Virginia Prostate Center of Eastern Virginia Medical School, Norfolk, Virginia, United States. Longitudinal prospective HRQoL survey. |
| Estape et al. 2009 | Patients with cervical cancer who underwent hysterectomy. Patients were matched to historical cohort based on stage and type of cancer. | Robotic radical hysterectomy | Laparoscopic radical hysterectomy and radical abdominal hysterectomy | Operative time (defined by the anesthesiologist as the time between the insertion of the foley catheter and the closing of the last trocar site. Blood loss, nodes retrieved, positive surgical margin rate, intraoperative and postoperative complications, transfusion rate, hospital stay, foley catherization days, days on pain medication, days to return to work, adjuvant treatment, alive or alive with disease at end of follow-up. | Most patients had their catheters removed on day two or three, however, this was not standardized. Follow-up consisted of visits at weeks 1 and 3 postoperatively then every 3 months for 2 years, followed by every 6 months until 5 years was completed. | Hospital: Patients undergoing robotic radical hysterectomy from August 2006-April 2008. Patients undergoing laparoscopic radical hysterectomy from July 2004-July 2006. Patients who underwent traditional radical abdominal hysterectomy from May 2002-July 2006. |
| Cardenas-Giocoechea et al. 2010 | Female patients undergoing minimally invasive total hysterectomy and pelvic and para-aortic lymphadenectomy | Robotic-assisted staging | Laparoscopic staging | Major intraoperative complications, including vascular injury, enterotomy, cystotomy, or conversion to laparotomy, were measured. Secondary outcomes including operative time, blood loss, transfusion rate, number of lymph nodes retrieved, the length of hospitalization, re-admission and re-operations rates were also measured. | At least 10 days post surgery. | Hospital: Traditional approach from January 2003-Deceber 2007, robotic-assisted approach from December 2007- July 2009. Study was conducted in an academic medical Center in the US (Pennsylvania Hospital). |
| Maggioni et al. 2009 | Patients with newly diagnosed invasive cervical cancer FIGO stages IA2, IB1, IB2 and IIA with no contraindications to surgery and no clinical or imaging (MRI and CT scan) evidence of nodal or parametrial involvement. | Robotic radical hysterectomy (RRH) | Abdominal radical hysterectomy (ARH) | Operating time (beginning of skin incision to completion of skin closure), estimated blood loss (difference in the total amounts of suctioned and irrigation fluids) complications intraoperative, early postoperative (<1 month after surgery) and late postoperative (>1 month after surgery). Length of hospital stay, discharge with or without catheter, bladder function 3 days after hysterectomy. | Voiding trial at postoperative day 3. Follow-up for complication at least 12 months. | Hospital: Procedures were performed at the European Institute of Oncology, Milan, Italy, between November 1, 2006 and February 1, 2009 |
| Sarlos et al. 2010 | Patients with benign lesions for whom vaginal hysterectomy was expected to be difficult because of large myomas or nulliparity and with an uterus weight estimated to be <500 g. | Robotic-assisted hysterectomy | Laparoscopic hysterectomy | Time for robot docking (time from when the robot was brought to the operating table until the surgeon start the operation at the console), total operating time (time from skin incision to the last skin closure suture), intra-operative complications, postoperative complications, dosage of postoperative analgesics, total postoperative hospital stay, personnel costs (calculated as costs per minute with different personnel factors based on salaries), material costs (materials used were recorded by medical staff and costs for purchase and sterilisation for reusable instruments were calculated), surgeons assessment of advantages, ergonomics and surgical aspects compared to laparoscopic surgery. | Surgeon questionnaire at the end of the procedure. Follow-up for postoperative outcomes at least until end of hospital stay (max 7 days). | Hospital: Kantonsspital Aarau, inclusion from June 2007 to May 2009 |
| Chang et al. 2012 | Patients with a clinical diagnosis of PAOD who visited the clinic volunteered to participate in this prospective study. PAOD was diagnosed by physical examination and confirmed by an angiogram, duplex ultrasonography, and ankle-brachial blood pressure index (ABPI). All patients received treatment with cilostazol before enrolling in the study. | The ArtAssist device (model AA-1000, ACI Medical, San Marcos, CA, USA) which has a frequency of 3 impulses/min, an impulse rise time of 1 s and a delay of 4 s for calf compression, was used. The inflation pressure of 120–140 mmHg. The deflation pressure was 0 mmHg, with a deflation time of 15 s. IPC was performed bilaterally in cases of bilateral claudication. IPC of the foot and calf was performed while the patient was seated. | Based on receipt of IPC therapy, patients were allocated to a study (n=23, IPC therapy positive) or control (n=8, IPC therapy negative) group. See (P) for inclusion criteria of both groups. | Self-assessment of QOL was evaluated with the SF-36 scoring system (translated into Taiwanese) by every patient at the beginning and the end of IPC therapy.  A 6-min walking test was performed for all patients at the beginning and end of IPC therapy. Initial and absolute claudication distances (ACDs) were recorded: the distance at which pain first occurred was the initial claudication distance (ICD) and the maximum distance terminated by pain was the ACD. The 6-min walking test was performed according to the statement guidelines for the American Thoracic Society.  The 6-min walking test, transcutaneous oxygen tension (TcPO2), and QOL evaluated with the Short-Form 36 questionnaire were measured at the beginning and end of the study. | Patients in the study group received IPC therapy for 3 h daily for 3 months. | The study was conducted in the Orthopedic clinic of the Chia-Yi Chang Gung Memorial Hospital, Taiwan. |
| Kavros et al. 2008 | Considered for inclusion were consecutive patients with chronic nonhealing toe or transmetatarsal amputation wounds and tissue loss of the foot attributable to chronic CLI,3 on whom all means of additional revascularization had been exhausted. Excluded were those with tissue loss of mixed cause (ie, venous, vasculitic, neuropathic, infective, traumatic); those who underwent alternative means of CLI treatment in the course of the study, including vasoactive pharmacotherapy (ie, prostaglandins) and epidural analgesia, as a means of enhancing their healing response to the tissue loss; those with deep vein thrombosis sustained =< 6 months of the study inclusion or during the study course, and finally, those with calf wounds. | Intermittent pneumatic compression of the calf was delivered using the ArterialFlow (DJO, Vista, Calif), a mechanical pneumatic pump consisting of a pneumatic impulse generator and a plastic inflatable pad (length, 29 cm; maximum circumference, 48 cm), specially designed to fit the calf. Large-bore elastic tubing connects the unit with the pad. The pump throughout the study was set to operate at a maximum inflation pressure of 85 to 95 mm Hg, delivered for 2 seconds with a rise time of 0.2 seconds, and a deflation pressure of 0 mm Hg of 18 seconds’ duration. A 24-hour help-line was offered for pertinent emergencies or medical advice. | A standardized wound care regimen for tissue loss and nonhealing amputation wounds of the foot due to chronic CLI, without the use of IPC. | Surgical outcomes at 18 months follow up: Survivorship, Complete healing (limb intact), BKA after a failed local amputation, Hemodialysis. | A minimum of 6 hours of IPC application per day, in three 2-hour sessions, was requested. Patient compliance was monitored by means of a recording device built-in the IPC pump and a patient logbook. As the IPC pumps were regularly inspected for optimal function, the IPC application time reported in the patient logbook was compared with that of the recording device. | A multidisciplinary wound-healing outpatient’s clinic comprising orthopedics, vascular surgery, and vascular medicine services. Performed in a community and multidisciplinary health care clinic (1998 through 2004); Mayo clinic USA. |
| Rogers et al. 2007 | Qualifying patients meeting the inclusion and exclusion criteria were enrolled in the LVAD or OMT arms of the trial based primarily upon patient preference or device availability. In practice, recruitment and study qualification was conducted on hospitalized patients with intermittent or continuous invasive hemodynamic assessments confirming the clinical impressions of disease status.  Eligible patients were adults with inotrope-dependent stage D heart failure, an ejection fraction of 25%, and New York Heart Association (NYHA) functional class IV symptoms for 3 months before enrollment and were not candidates for cardiac transplantation based upon site-specific transplant program criteria. Patients had been treated with maximally tolerated doses of angiotensin-converting enzyme inhibitors, beta-blockers, digoxin, diuretics, and/or other vasodilators. All patients were receiving inotropic therapy for clinical and/or hemodynamic evidence of circulatory failure. Before enrollment, the inotropic drugs were weaned on 2 separate occasions separated by at least 7 days. Inability to successfully wean from inotropes was defined by at least 1 of the following: systemic hypotension, exacerbation of heart failure symptoms, worsening end-organ function, a cardiac index of 2.2 l/min/m2, or a pulmonary capillary wedge pressure of 20 mm Hg. | The Novacor LVAD is a surgically implanted 1 kg electromagnetically actuated blood pump capable of outputs up to 10 l/min. A conduit placed in the left ventricular apex diverts blood into the pump, and the pump returns blood to the ascending aorta. A driveline tunneled across the abdominal wall is connected to a wearable controller (0.6 kg) that regulates and monitors pump function and a pair of portable batteries (2.7 kg) that provide uninterrupted power for 6 h. | An optimal medical therapy (OMT) treatment strategy. | The primary end point of the INTrEPID trial was all-cause mortality at 6 months. Secondary end points included adverse events, functional capacity, and healthrelated quality of life. | Primary endpoint measured at 6 months | The INTrEPID trial was a nonrandomized, 2-arm clinical trial conducted at 13 centers in the U.S. and Canada with experience implanting the Novacor LVAD as a bridge to transplantation. The trial was approved by the U.S. Food and Drug Administration (FDA) as a feasibility study.  The costs of data collection were covered by WorldHeart. Patient care costs were shared by the manufacturer and the patients’ public or private insurance as a condition of device availability. An independent Study Steering Committee approved the study design, and a Data and Safety Monitoring Board followed the conduct of the trial and adverse events.  Patients provided informed consent before enrollment, and the Institutional Review Board at each of the participating institutions approved the trial. |
| Bestehorn et al. 2015 | Based on the 2013 AVR datasets of the Federal Joint Committee - see S. Patients with transapical aortic valve implantation were excluded due to substantial differences in basic data like profile of co-morbidities and cardiac function (e.g. low cardiac output: 10% vs. 5% in SAVR patients and 2% in TAVI patients) compared to TF patients. Two groups of patients were compared: patients treated by isolated SAVR and those with (isolated) TF. In the TF group (n¼7620) 48 procedures were performed with EC and in 22 cases a switch to EC was necessary.   Patients with EuroScore between 10% and 20% were extracted from the database of all isolated AVR procedures in 2013. Overall 3407 cases, 771 SAVR patients and 2636 TF patients were available for analysis. | The surgical aortic valve replacement (SAVR) | The transfemoral approach (TF) is used as an alternative for high risk symptomatic patients who in principle would be eligible for SAVR. | The instruction for documentation of the AVR dataset does not contain any information about assessment of POD and differentiates only between POD and POD with need for therapy (POD-T). In order to focus on clinically relevant and valid parameters POD with need for therapy and in-hospital mortality were chosen as target parameters.  In-hospital mortality and POD-T. | Retrospecive review from 2013 AVR databases. | This analysis is based on the 2013 AVR datasets of the Federal Joint Committee, held by the AQUA Institute (Gottingen, Germany), which were documented in accordance with x137 Social Security Code V (SGB V) by hospitals registered under x108 SGB V. Data collection is compulsory for all in-patient procedures in hospitals billing AVR to German statutory health insurance or private insurance companies (95 hospitals). The quality of the dataset is controlled by a validated system including testing for plausibility and correctness and, in case of statistical outliers, structured interviews. |
| Brennan et al. 2017 | Detailed clinical information and Medicare claims-based followup were available for 25,786 TAVR cases performed between January 1, 2014, and September 30, 2015, and 198,077 SAVR (or SAVR plus coronary artery bypass grafting) cases performed between July 1, 2011, and December 31, 2013. Patients with characteristics that were thought to strongly favor 1 treatment or another were excluded. Following these exclusions, the population of interest included 17,910 TAVR and 22,618 SAVR patients who were available for propensity matching. | Transcatheter aortic valve replacement (TAVR) | Surgical aortic valve replacement (SAVR) | Primary outcomes of interest were chosen by consensus and included death, stroke, days alive and out of an acute care hospital facility (i.e., days alive and out of the hospital [DAOH]) to 1 year, and discharge home. Stroke and mortality were evaluated to 30 days and 1 year over a median follow-up period of 169.5 days for TAVR and 328 days for SAVR. Stroke was identified during the index procedural hospitalization using registry data. Following hospital discharge, stroke was identified using Medicare rehospitalization claims with a primary position International Classification of Diseases, Ninth Revision, Clinical Modification code of 434.x1, 436, 433.x1, 997.02, 437.1, 437.9, 430, 431, or 432.x. | The availability of ResDAC files generally lags 12 to 18 months behind the date of service provision. | Data for this analysis were drawn from 2 U.S. procedural registries: 1) SAVR data were drawn from the Society of Thoracic Surgeons (STS) National Database; and 2) TAVR data were drawn from the STS/American College of Cardiology TVT (Transcatheter Valve Therapy) Registry. More than 90% of cardiac surgery programs in the United States participate in the STS National Database, and participation in the TVT Registry is necessary for Medicare reimbursement. Notably, the involvement of a heart team is also necessary for Medicare reimbursement in the United States. |
| Thanopoulos et al. 2006 | Ninety-two (92) consecutive patients with a PFO and at least one documented TIA or stroke of recent origin (<30 days) were enrolled in this study. Patients were divided into two groups on the basis of a nonrandomized, patient preference case series protocol: Group I consisted of 48 patients (male/female 27/21, mean age 43 6 11) who underwent transcatheter closure of the defect using the Amplatzer PFO occluder (APFOO). Patients were selected according to a previously agreed protocol with full and informed consent. All devices were implanted in the context of the study protocol approved by the Ethical Committees of the participating Hospitals. Group II consisted of 44 patients (male/female 21/23, mean age 40 6 12) who had declined catheter treatment and received long-term treatment with clopidrogel (75 mg/day) and aspirin (325 mg/day). | The APFOO (AGA Medical, Golden Valley, MN) is constructed from nitinol wires and consists of two flat disks with a thin connecting waist 3 mm in length [22]. The right atrial disk is larger than the left atrial disk. At the time of the study, the device was available in two sizes, 25 and 35 mm, respectively. It should be noted that the APFOO is approved for clinical use device in Europe. (EEC certificate: 199C/DE).  Postinterventional treatment included oral aspirin (100 mg once a day) for 6–9 months and clopidogrel (75 mg once a day) for 6 months. To prevent infectious complications, amoxicillin (500 g bid) was given for 3 days. All patients were sent home the next day after the procedure. Prophylaxis against infectious endocarditis was recommended for 6 months according to the guidelines of the American Heart Association. | Long-term treatment with clopidrogel (75 mg/day) and aspirin (325 mg/day). | Analyses of the data included the description of periinterventional event rates and complications as well as the complications during the follow-up period. | A transthoracic echocardiography study was performed within the first few days after transcatheter PFO closure. All patients were followed-up prospectively for up to 24 months. After percutaneous PFO closure, clinical examinations were carried out at 1, 6, 12, and 24 months, including detailed neurological and medical examination, a 12- lead-ECG and a transthoracic echocardiogram (TTE). A TEE with contrast bubble study at rest and during Valsalva maneuver was performed 6 months after the invasive procedure in group I. Patients with suspected recurrent thromboembolic events were reevaluated by their neurologist and additionally by cerebral CT and/or MRI. | Department of Cardiology, ‘Aghia Sophia’ Children’s Hospital, Athens, Greece Department of Cardiology, Thessaloniki Heart Institute, ‘Agios Loukas’ Hospital, Thessaloniki, Greece |
| Windecker et al. 2004 | We identified all patients with transient ischemic attack (TIA) or ischemic stroke, who were admitted to our university hospital stroke center between January 1994 and August 2000. After excluding patients without a PFO or with a concurrent etiology for the cerebrovascular event, 308 patients were classified as having suffered a cryptogenic stroke presumably related to PFO. Percutaneous PFO closure was performed in 150 patients, whereas 158 patients were treated medically. The individual treatment decision was based on patient and physician preference. | Percutaneous PFO closure. The procedure was performed under local anesthesia and fluoroscopic guidance as Six different device types were utilized according to availability at different time points, including the Amplatzer PFO Occluder (n = 54), PFO STAR (n = 42), Sideris buttoned device (n = 27), Angel Wing device (n = 10), Amplatzer ASD Occluder (n = 9), and CardioSEAL (n = 8). Patients were treated with acetylsalicylic acid 100 mg once daily for six months for antithrombotic protection until full device endothelialization. | Patients assigned to medical treatment were treated with a vitamin K antagonist or antiplatelet therapy at the discretion of the attending neurologist. Coumadin was adjusted to a target international normalized ratio of 2.0 to 3.0; acetylsalicylic acid was prescribed at a mean dose of 233 83 mg/day, and clopidogrel at a dose of 75 mg/day. | The main outcome was the occurrence of cerebrovascular index events (Transient ischemic attack/ Ischemic stroke). Other outcomes included number of previous events. | A contrast transesophageal echocardiography was repeated six months after percutaneous PFO closure to assess for a residual shunt after endothelial overgrowth. Medical treatment was discontinued in 93 of 150 patients after six months. Fifty-seven patients continued medical treatment with acetylsalicylic acid 100 mg once daily owing to a residual shunt (26 patients) or presence of mild coronary artery disease as assessed by coronary angiography (31 patients).  All family physicians and patients were subjected to a structured interview addressing recurrence of cerebrovascular events, rehospitalizations, and device- or medication-related problems. Death, recurrent ischemic stroke, TIA, or peripheral embolism were considered adverse events. Patients with suspected recurrent cerebrovascular events were reexamined by a neurologist, and an imaging study of the brain was repeated. Follow-up information was available for all patients at some point in time, but two patients in the medical treatment group and three patients in the percutaneous PFO closure group were subsequently lost to follow-up as a result of address changes. Patients gave informed consent, and the study was approved by the local ethics committee. | University hospital in Bern. Study period: 1994 to 2000 |
| Schuchlenz et al. 2005 | Patients with cryptogenic cerebrovascular events and a patent foramen ovale | Cathether device closure | Platelet inhibitors or anticoagulation | Recurrent events, death and severe treatment complications | Patients were followed until an endpoint was reached. In the absence of a recurrent event, the follow-up was completed in all event-free survivors in February 2002. Mean follow up was 2.6 years | Hospital: A routine clinical setting in Austria (1992-2002) |
| Harrer et al. 2006 | Patients with cryptogenic cerebral ischemia and patent foramen ovale | Invasive treatment (transcatheter closure or surgical closure) | Medical treatment (antiplatelet or anticoagulant therapy) | Recurrent ischemic events | Mean follow up was 4.3 years | Hospital: Two neurological departments in Germany |
| Casaubon et al. 2007 | Patients with cryptogenic stroke or TIA and patent foramen ovale | Percutaneous device closure or surgical closure | Antiplatelet agents or anticoagulation | Recurrent stroke and a composite of recurrent stroke, TIA, and death from vascular causes | Mean follow up was 2.7 years | Hospital: A large acute care hospital in Ontario (1997-2003) |
| Sherfey et al. 2007 | Patients who had a primary total hip arthroplasty (mainly patients with degenerative arthritis) | Exeter cemented stem | Endurance cemented stem | Revisions for any reason and for aseptic loosening of the femoral stem | Mean follow up was 7.25 years (Exeter stem) and 4.57 years (Endurance stem) | Hospital: single surgeon. From 1994 to 2000, 118 patients representing 138 hips were implanted with the Exeter stem. From 1997 to 2000, the Endurance stem was implanted in 41 patients. |
| Bozic et al. 2005 | Patients who had total knee arthroplasty with a third-generation cemented prosthetic device | Posterior cruciate-retaining total knee arthroplasty with the NexGen cruciate-retaining prosthesis (Zimmer) | Posterior-stabilized total knee arthroplasty using the NexGen Legacy posterior-stabilized prosthesis (Zimmer) | Revision for any reason and revision for aseptic loosening, reoperation rates, and complication rates | Five to eight years follow-up | Hospital in the US. Between July 1995 and July 1997, 334 consecutive primary TKAs were done in 287 patients . |
| Furnes et al. 2002 | Patients who had total knee replacements | Comparison between the prosthesis brands, their types of fi xation, and whether or not the patella was resurfaced. | - | Revision for all causes | Zero to 6.5 years | Hospitals in Norway; primary total knee replacements reported to the Norwegian Arthroplasty Register, operated on between 1994 and 2000. |
| Schuijf et al. 2006 | Patients with an intermediate likelihood of coronary artery disease (CAD) | Multi-slice computed tomography (MSCT) | Myocardial perfusion imaging | The MSCT studies were classified as having no CAD, nonobstructive (<50% luminal narrowing) CAD, or obstructive CAD. Myocardial perfusion imaging examinations were classified as showing normal or abnormal (reversible and/or fixed defects). | In addition to myocardial perfusion imaging, patients underwent noninvasive coronary angiography with MSCT within 1 month. | Hospital: Two outptient clinics in The Netherlands and Belgium |
